# Willingness of caregivers to have their daughters vaccinated against human papilloma virus and associated factors in Jimma Town, Southwest Ethiopia

**DOI:** 10.3389/fgwh.2024.1400324

**Published:** 2024-12-12

**Authors:** Anebo Getachew, Susan Anand, Tilahun Wodaynew

**Affiliations:** ^1^ Midwifery Department, Hosanna College of Health Sciences, Hossana, Ethiopia; ^2^ School of Nursing, Jimma University, Jimma, Ethiopia; ^3^ Department of Nursing, College of Health Science, Woldia University, Woldia, Ethiopia

**Keywords:** human papillomavirus vaccine, cervical cancer, caregiver's willingness, Jimma, Ethiopia

## Abstract

**Introduction:**

Human Papillomavirus (HPV) is a widespread sexually transmitted infection and a leading cause of cervical cancer. Although there is a significant HPV prevalence in Ethiopia, yet the uptake of the HPV vaccine remains low. This study aimed to assess the level of caregivers' willingness to vaccinate their daughters against the human papilloma virus and associated factors in Jimma town.

**Methods:**

A community-based cross-sectional study was conducted from June 1–30, 2023. A total of 471 study participants were selected using multi-stage sampling techniques. Data was collected using an interviewer-administered questionnaire. Binary and multiple logistic regression analyses were done to identify associated factors, and the adjusted odds ratio and 95% confidence interval were computed. A value *p* of <0.05 was used to determine statistical significance.

**Results:**

About 82.4% (95% CI: 79.0–86.0) of caregivers indicated a willingness to have their daughters vaccinated. Having college education or above (AOR:3.31, 95%CI:1.02–10.8), good knowledge of the HPV vaccine (AOR:2.25, 95%CI:1.05–4.85), good knowledge of sexually transmitted infections (STIs) (AOR:2.04, 95%CI: 1.09–3.82), good knowledge of cervical cancer (AOR:2.50, 95%CI:1.31–4.77) and a positive attitude towards the vaccine (AOR:4.03, 95%CI: 2.26–7.22), were associated with willingness.

**Discussion:**

The majority of caregivers were willing to vaccinate their daughters against HPV. Caregivers who had higher education, good knowledge about the HPV vaccine, cervical cancer, and STIs, as well as positive attitudes towards the vaccine, were more likely to be willing to vaccinate. Efforts should be made to educate caregivers about the vaccine, cervical cancer, and STIs while promoting positive attitudes.

## Introduction

Human papillomavirus (HPV) is a virus that can infect the skin and mucous membranes in the nose, throat, and genitals. It causes malignancies in the cervix, anus, penis, oropharynx, and vagina (1). The percentage of women with HPV varied in different parts of the world, ranging from 3% in Australia/New Zealand and the United States to 26% in sub-Saharan African countries (SSA) in 2017 (2). In 2022, countries in the SSA had an HPV prevalence rate of 34% (3). High-risk HPV types, such as 16, 18, and others, are associated with 99% of cervical precancers globally (4). The fourth most frequent cancer in women worldwide is cervical cancer. In 2020, low- and middle-income countries accounted for almost 90% of global new cases and fatalities (5). Globally, 604,000 new cases of cervical cancer and 342,000 deaths were reported in 2020 and SSA countries have the greatest rates of regional incidence and mortality, with particularly high rates in Eastern Africa (6). The rising prevalence of HPV infections and the burden of cervical cancer in Ethiopia make it a public health concern. In Ethiopia, cervical cancer is the second-most common type of cancer and there are 36.9 million women who are at risk of cervical cancer. In Ethiopia, statistics showed that 7,445 women are diagnosed with cervical cancer each year, and 5,338 of them die from the adisease (7, 8).

HPV vaccination, pre-cancer screening, and treatment of pre-cancerous lesions are all cost-effective interventions that can significantly reduce the burden of cervical cancer (9–11) However, for several reasons, public acceptance has lagged (12, 13). The World Health Organization (WHO) recommends a one- or two-dose schedule of HPV vaccination for girls aged 9–14 and 15–20 years and two doses for women over 21 years. Girls between the ages of 9 and 14 are the main target of immunization (14). The coverage of the first HPV vaccine dose decreased from 25% to 15% between 2019 and 2021. As a result, 3.5 million more girls failed to receive vaccines (14). Despite the HPV vaccine being accessible for years, dosage completion in SSA countries is still as low as 20% (15). The HPV vaccine was introduced in Ethiopia in December 2018 with a focus on 14-year-old girls with the support of the Global Alliance for Vaccines and Immunization (GAVI) (16). Despite the fact that the Ethiopian Federal Ministry of Health (17) has been working on several preventative and treatment measures, including integrating cervical cancer screening and treatment into healthcare services for women (18), the HPV vaccine uptake in Ethiopia is much lower than the WHO goal. This shows a large gap that needs to be closed to reach the WHO's goal of eliminating cervical cancer (19, 20). Caregivers' willingness to have their children vaccinated is critical to the uptake of the HPV vaccine since the success of vaccination depends on caregivers’ willingness to vaccinate their daughters. To reach all eligible girls in Ethiopia, the vaccine is predominantly administered through a school-based strategy (16). Studies conducted in Ethiopia assessed several factors that affect parental willingness. However, factors like the father's approval for vaccination, knowledge of STIs, and health beliefs regarding cervical cancer were not studied, which may influence caregivers' willingness and there was no study conducted in this study area. So, this study examined the association of those factors with the caregivers' willingness to vaccinate their daughters in Jimma town, southwest Ethiopia.

## Materials and methods

### Study area and period

The study was carried out in Jimma Town, which is Jimma Zone's capital city. It is one of the towns in the regional state of Oromia region in southwest Ethiopia, located 352 kilometers from Addis Ababa. It is located at a height of roughly 1,780 meters above sea level (21). For administrative reasons, the town is divided into 17 kebeles. In the 2015 Ethiopian calendar, the total population of the town was 241,372, according to the data obtained from the Jimma town health office (unpublished). One teaching specialized hospital (Jimma Medical Center), one primary hospital (Shanan Gibe General Hospital), five health centers, and numerous private clinics are providing health care services to the population of the town (22). The study was conducted from June 1–30, 2023.

### Study design

A community-based cross-sectional study was employed.

### Population

All caregivers in Jimma town with daughters in the age range of 9–14 years were the source of population, while all caregivers having daughters in the age group of 9–14 years in the selected Kebeles were considered as study populations. Caregivers having daughters in the age group of 9–14 years who lived permanently in the study area (for more than 6 months) were included in the study.

### Sample size determination

The sample size was calculated by using OpenEpi Info version 7.2 software for each specific objectives. Accordingly, the largest sample size of the objective was taken as the final sample size of the study. Therefore, the sample size for this particular study was 471 individuals.

### Sampling procedure

The study participants were selected using a multistage sampling method. First, Keble was selected using a simple random sampling technique. Among a total of 17 kebeles in Jimma town, 30% (5 kebeles) were selected. Second, a systematic random sampling technique was used to select households with daughters aged 9–14 from the selected kebele by considering proportional allocation. The sampling interval was determined by dividing the total households with daughters aged 9–14 from the selected kebele by the total sample size. The first participant was selected randomly by a lottery method from 1 to 7, and the next respondent was chosen at regular intervals (every 7th household). When there were two caregivers from each household, participants were informed that one of them could provide information and selected by lottery method for the study. On the condition that study participants were not available at the first visit, this household was revisited once on the same day or the following day. If not available again, the study participant was considered a non-respondent.

### Data collection tools and procedures

Data were collected by the structured interviewer-administered questionnaire that was developed after an extensive review of relevant literature (23–31). The tools contained structured questions in nine sections: sociodemographics, knowledge of HPV and HPV vaccine, knowledge of cervical cancer, knowledge of STIs, attitude towards HPV vaccination, reproductive health characteristics, health beliefs towards cervical cancer, father consent, and caregiver willingness for HPV vaccination. Ten health extension workers were recruited as data collectors, with two supervisors who could speak local languages. Each interview was carried out in a private, quiet setting within the home and lasted approximately 20 min.

### Study variables

#### Dependent variable

• Caregiver's Willingness.

#### Independent variables

Socio-demographic variables, knowledge-related factors, approval of fathers, attitude, reproductive health-related factors and health beliefs towards cervical cancer.

#### Measurement and definition of terms

##### Knowledge

For each questionnaire, overall knowledge was determined by summing up the total number of correct responses and comparing that to a defined threshold; answering above the seted threshold indicated good knowledge, while answering below denoted poor knowledge. The HPV infection questionnaire had 8 questions. Participants who answered more than four questions correctly were categorized as having good HPV knowledge; otherwise considered to have poor knowledge (32). The 5-question HPV vaccine questionnaire required participants to answer at least 3 correctly to be denoted as having good knowledge, otherwise indicating poor knowledge. For the 7-question cervical cancer questionnaire, more than 3 correct answers represented good knowledge, otherwise poor knowledge (32). Finally, the 22-question STI knowledge assessment categorized participants as having good knowledge of STI if they were answered more than 11 questions correctly and poor knowledge if they answered 11 or fewer correctly (32).

##### Attitude

A five-point Likert scale was used to assess attitude, where various degrees of attitude would be assessed using options of (1) strongly disagree; (2) disagree; (3) undecided; (4) agree; and (5) strongly agree. The individual attitude scores were summed for each participant and then converted into a percentage value. An attitude percentage score between 50.0% and 100.0% was categorized as a positive attitude. An attitude percentage score of less than 50.0% was categorized as a negative attitude (33).

##### Health beliefs towards cervical cancer

Participants were awarded different point values based on their answers to a set of questions: the first question allowed scores of 0 to 4, the second and third questions allowed for 0 or 1 point each, and the fourth question had possible scores of 0 to 3. The total scores were calculated by summing these points, and the resulting score was converted into percentages. Respondents who had a score of 50% to 100% were categorized as having cervical cancer health beliefs in favor of HPV vaccination, while less than 50% were against HPV vaccination (33).

Willingness: was categorized as “Yes” or “No”, with a “Yes” response indicating the caregiver's willingness to vaccinate their daughter(s) against HPV (24, 34).

Caregiver: is a parent or guardian who has the authority and corresponding duty to care for the personal and property interests of a daughter (35).

### Data quality assurance

To ensure the quality of the data gathered from the study subjects, a range of mechanisms were employed. The questionnaire that was initially prepared in English was translated into Afan Oromo and Amharic. The pre-test for the study was conducted with 5% (23 participants) of the total sample size in Seto Semero kebele, which was similar in population characteristics. The questionnaire's knowledge and attitude sections had Cronbach's alpha values of 0.8 (HPV and HPV vaccine), 0.78 (knowledge of cervical cancer), 0.71 (Attitude towards HPV vaccination), and 0.7 (knowledge of STIs), which suggested that the reliability of the questionnaire was acceptable. Participants' feedback helped to refine and clarify any ambiguous survey questions. Data collectors and supervisors were oriented for one day about the objective, tools, and process of data collection. The supervisors completed day-to-day on-site supervision and carried out on-site checks and reviews of all completed questionnaires.

### Data processing and analysis

Data were entered into Epidata version 4.6. Statistical Software for Social Sciences (SPSS) version 25 was used to analyze the data. Descriptive statistics like frequencies, percentages, means, and standard deviations were used to examine the data. The Hosmer-Lemeshow test was used to assess the goodness of fit of the model, and the result was not significant, with a *p*-value of 0.707. This non-significant *p*-value suggests that the model adequately fits the data. A multicollinearity test between independent variables was checked by using the variance inflation factor and tolerance test. The VIF and tolerance test values for all of the independent variables were less than 2 and greater than 0.5 respectively, which were generally considered to be acceptable. Binary logistic regression was used to identify candidate variables for multiple logistic regressions at a *P*-value < 0.25. The factors that were associated with willingness to vaccinate their daughter against HPV were declared using a cut-off *P* < 0.05 in multiple logistic regression analysis. Data were presented by using tables, graphs, and texts.

### Ethical consideration

The study received ethical clearance from the Institutional Review Board of Jimma University, with reference number JUIH/IRB/404/23. Permission was also obtained from the Jimma City Administration Health office. Participants were fully informed about the purpose and nature of the study, and written and verbal informed consent was obtained from each participant before enrollment. The confidentiality of the information was maintained through anonymous questionnaires. Participants were assured that their personal information would remain confidential and would not be shared outside of the study team.

## Result

### Socio-demographic characteristics of the study participants

This study had a total of 459 participants, which resulted in a high response rate of 97.45%. The mean age of respondents was 36.43 years (SD ± 6.513). Out of the total respondents, 270 (58.8%) were female. The majority were married 405 (88.2%) and 159 (34.6%) had attended college or higher. Majority of households 169 (36.8%) had low monthly income (<3,000 ETB). Out of a total of 459 households, 285 (62.1%) had their daughters enrolled in government schools. Additionally, 412 households (89.8%) had only one daughter (Table 1).

**Table 1:** Sociodemographic characteristics of the study participants surveyed in jimma town, southwest Ethiopia, 2023. (*N* = 459).

Variables	Category	Frequency (%)
Age	≤29	70 (15.3%)
	30–39	236 (51.4%)
	≥40	153 (33.3%)
Sex	Male	189 (41.2%)
	Female	270 (58.8%)
Current marital status	Single	8 (1.7%)
	Married	405 (88.2%)
	Divorced	26 (5.7%)
	Widowed	20 (4.4%)
Educational status	No formal education	22 (4.8%)
	Primary education	131 (28.5%)
	Secondary education	147 (32.1%)
	College and above	159 (34.6%)
Average monthly income	Low income (<3,000 ETB)	169 (36.8%)
	Medium income (3,000–7,500 ETB)	152 (33.1%)
	High income (>7,500 ETB)	138 (30.1%)
The school type that the daughter enrolled	Government School	285 (62.1)
	Private school	174 (37.9)
Number of daughters aged 9–14	One	412 (89.8)
	More than one	47 (10.2)

### Fathers consent

The majority 381 (83%) of respondents indicated that the father's approval is not mandatory for the vaccination of daughters.

### Knowledge about HPV infection and HPV vaccine

**Figure 1:**
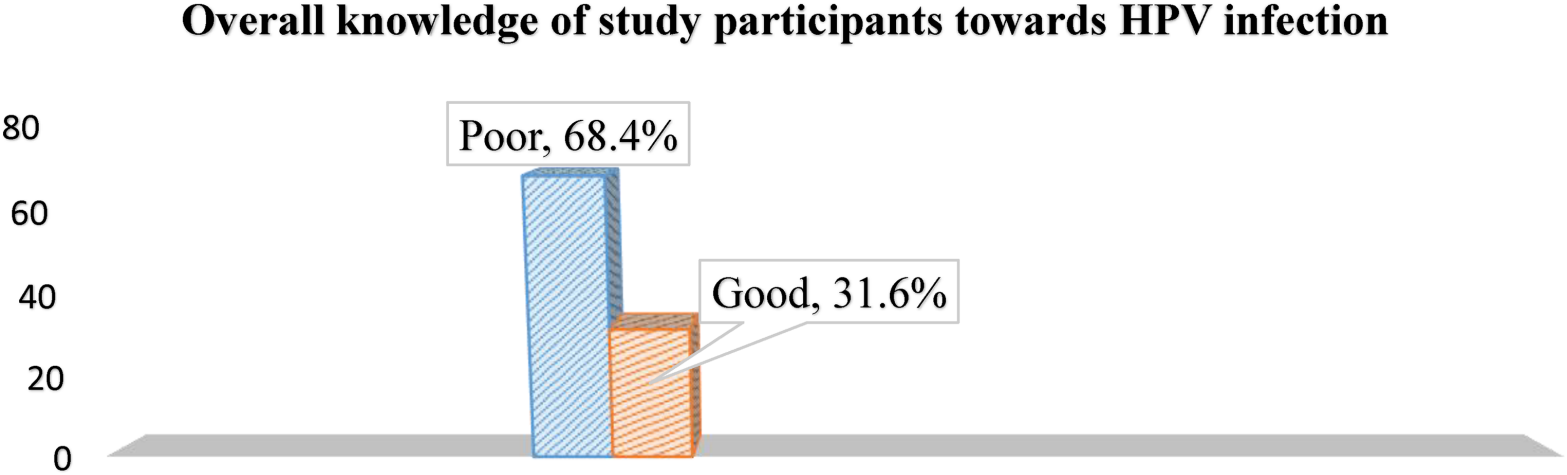
The overall knowledge of study participants about HPV infection in jimma town, southwest Ethiopia, 2023.

**Figure 2:**
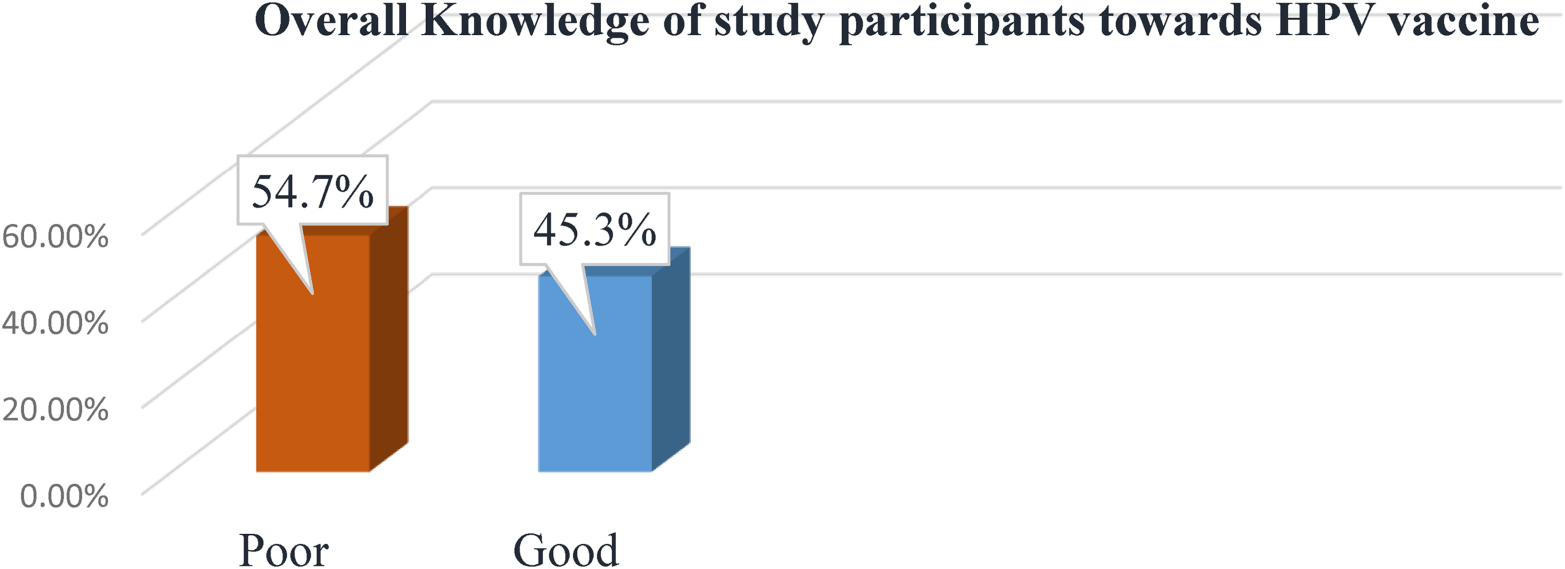
The overall knowledge of study participants about HPV vaccine in jimma town, southwest Ethiopia, 2023.

This study assessed the overall knowledge regarding HPV infection and HPV vaccine among participants described below, respectively (see Figures 1, 2).

### Knowledge about cervical cancer and STI

The majority of respondents have heard of cervical cancer and cervical cancer screening. Out of the total 459 participants, over half 270 (58.8%) demonstrated good knowledge of cervical cancer. The remaining participants 189 (41.2%) exhibited poor knowledge of cervical cancer. The majority of caregivers mentioned common STIs like HIV, gonorrhea, and syphilis. From STI symptoms, the most mentioned were genital ulcers and genital discharge. The study found that a majority of caregivers 269 (58.6%) had good knowledge of STIs, while the remaining participants 190 (41.4%) had poor knowledge.

### Attitude of study participants towards the HPV vaccine

This study assessed attitudes toward HPV vaccination among 459 respondents. The results revealed that a majority of respondents 329 (71.7%) had a positive attitude regarding HPV vaccination, while only 130 (28.3%) had a negative attitude towards HPV vaccination.

### Level of the willingness of caregivers to have their daughters vaccinated against HPV

This study revealed that the majority of respondents 378 (82.4%) (95% CI, 79.0–86.0) expressed willingness to have their daughters vaccinated against HPV.

### Factors associated with the willingness of caregivers to have their daughters vaccinated against HPV

In multiple logistic regression analysis, the variables showed that higher education levels, good knowledge of the HPV vaccine, a positive attitude towards the vaccine, good knowledge of STIs, and good knowledge of cervical cancer were more likely to be associated with a willingness to vaccinate daughters (Table 2).

**Table 2:** Binary and multivariable logistic regression analysis to select associated variables in jimma town, southwest Ethiopia, 2023 (*N* = 459).

Variable	Category	Willingness		COR (95% CI)	AOR (95%CI)	*p*-value
		No	Yes			
Educational status	No formal education	7	15		1	
	Primary education	29	102	1.64 (0.611–4.406)	1.89 (0.60–5.92)	0.28
	Secondary education	26	121	2.172 (0.81–5.86)	2.06 (0.65–6.52)	0.22
	College and above	19	140	3.44 (1.24–9.51)	3.31 (1.02–10.8)	0.047*
Knowledge of respondents towards HPV Vaccine	Poor	68	183		1	
	Good	13	195	5.57 (2.98–10.43)	2.25 (1.05–4.85)	0.038*
Attitude of respondents	Negative	47	83		1	
	Positive	34	295	4.91 (2.97–8.13)	4.03 (2.26–7.22)	0.00*
Knowledge of respondents towards STI	Poor	57	133		1	
	Good	24	245	4.36 (2.6–7.37)	2.04 (1.09–3.82)	0.025*
Knowledge of respondents towards Cervical cancer	Poor	61	128		1	
	Good	20	250	5.96 (3.44–10.30)	2.50 (1.31–4.77)	0.005*
Knowledge of respondents towards HPV infection	Poor	74	240		1	
	Good	7	138	6.08 (2.72–13.6)	1.15 (0.43–3.06)	0.780
Number of daughters aged 9–14	One	68	344		1	
	More than one	13	34	0.52 (0.26–1.03)	0.43 (0.18–1.01)	0.052
Health beliefs towards Cervical cancer	Unfavorable	6	6		1	
	Favorable	75	372	4.96 (1.56–15.8)	1.74 (0.48–6.28)	0.397
Fear of HPV	No	10	21		1	
	Yes	71	357	2.39 (1.08–5.30)	1.98 (0.79–4.97)	0.148

* = *P* ≤ 0.05.

## Discussion

The current study found that 82.4% of caregivers were willing to vaccinate their daughters against HPV. This rate is higher than the willingness reported in Korea (70%), China (73.9%), France (37.8%), and Ilorin, Nigeria (44.9%) (26, 36–38). The variation in HPV vaccine willingness rate observed in the current study may be due to the 5–8year gap between the current study and previous studies. This gap may provide time for changes to have occurred in public awareness, social norms, government policies, and vaccine availability, all of which can affect HPV vaccine willingness. The cost of the HPV vaccine had an impact on caregivers' willingness to get their daughters vaccinated (39).

The current study finding (82.4%) is slightly lower than the willingness in the other Nigerian study (89.1%), the Tanzania study (93%), and the Brazilian study (92%) (40–42). The differences in vaccination programs between countries might be a possible reason. Vaccination policies and recommendations varied between countries, impacting the willingness. For instance, Brazil introduced HPV vaccination into their national immunization program in 2014 (43). Another potential explanation for the disparities observed could be attributed to variations in socio-demographic factors among study participants between the current study and Nigerian and Brazilian studies. Specifically, the percentage of respondents lacking formal education was higher in the current study (4.8%) compared to the Nigerian study (4.1%) and the Brazilian study (1%). This is because educated caregivers were more likely to have been exposed to information about the HPV vaccine and cervical cancer through sources such as the media, the internet, and health campaigns, which influence caregivers' willingness (44). The level of willingness of caregivers to vaccinate their daughters against HPV in the current study was moderately low compared to studies done in Addis Ababa (94.3%) (35). The discrepancy might be that Addis Ababa is more cosmopolitan, with higher education levels and socioeconomic status. Addis Ababa has greater access to HPV vaccine awareness campaigns and health information, leading to higher knowledge levels, which contribute to increased willingness. The other reason for the discrepancy might be the Addis Ababa studies had a larger proportion of participants with good HPV knowledge (60.1%) compared to the current study (45.2%). Increased awareness of the HPV vaccine likely contributes to a higher caregiver willingness to vaccinate daughters (45).

In contrast, the willingness documented in Debre Tabor was markedly lower than the current study finding, with only 44.8% of parents willing to vaccinate their daughters against HPV (23). The possible reason might be that educational or promotional efforts related to the HPV vaccine took place during those 2 years, influencing the willingness. The other probable reason might be that the Debre Tabor study was conducted during the COVID-19 pandemic, which likely lowered willingness due to health system disruptions at that time (46, 47). The educational status difference of the study participants could be another reason for the discrepancy. This is because educational status plays a crucial role in enhancing health literacy and promoting understanding of preventive measures, such as vaccinations (44).

The current study findings showed that higher education levels were associated with an increased willingness to vaccinate daughters against HPV. This finding is congruent with the study done in Nigeria in different districts (48, 49). Studies in Ethiopia in Bench Sheko and Debre Tabor also showed higher education levels are associated with increased willingness to vaccinate daughters against HPV (23, 34). The possible explanation could be that highly educated caregivers are more likely to vaccinate their daughters against HPV because they have greater health literacy and understand the benefits of vaccination better (50).

A current study has found that good caregivers' knowledge of the HPV vaccine is key to improved willingness. This finding is consistent with multiple studies in countries including the United States, Thailand, China, and Kenya (37, 51–53). The Debre Tabor, Addis Ababa, and Bench Shako studies also demonstrated participants with good HPV vaccine knowledge were more willing to vaccinate daughters (23, 34, 35). This is probably because increased HPV vaccine knowledge leads to a better understanding of the benefits of the vaccine, reduces vaccine hesitancy, and empowers caregivers to make informed decisions about their daughters' health (54).

The positive association between caregivers' good cervical cancer knowledge and increased HPV vaccine willingness found in the Jimma study were mirrored findings from prior studies in Nigeria (48, 49). It also aligns with the Gondar study (24). This could be the fact that those who have a better knowledge of cervical cancer are more aware of the risk factors, disease severity, and benefits of the vaccine and view vaccination as a way to protect their daughters from cervical cancer.

The current study in Jimma found that caregivers with positive attitudes towards HPV vaccination were more likely to be willing to vaccinate their daughters compared to those with negative attitudes. This aligns with previous studies globally, including in Poland, Argentina, Austria, and Kenya (25, 44, 55, 56), and nationally in Addis Ababa, Gondar, and Bench Sheko (24, 34, 35). The possible explanation could be caregivers' views and opinions about the efficiency of the recommended HPV vaccine, which play a big role in vaccine acceptance. Another possible explanation could be the widespread belief that cancer is a serious and fatal disease (49).

The current study in Jimma found that caregivers' good knowledge regarding STIs was associated with increased willingness to vaccinate daughters against HPV compared to poor STI knowledge. This aligns with an earlier study in Nigeria (57). A possible explanation could be that caregiver health literacy regarding STIs predicts preventative behaviors. Those with more knowledge are more proactive about protecting their daughters via vaccination. Unlike most previous studies examining willingness in Ethiopia, the current study in Jimma provides new evidence on the relationship between caregiver knowledge of STIs and their willingness to vaccinate daughters against HPV in the Ethiopian context.

### Limitations of the study

Self-reported data may be subject to recall or social desirability biases. However, to reduce such bias, participants were first fully informed about the study's aim and data confidentiality procedures.

### Strengths of the study

This study contributes novelty to the field. Unlike previous studies focused on willingness in Ethiopia, the current study conducted in Jimma offers fresh evidence regarding the connection between caregiver knowledge of STIs and their willingness to vaccinate their daughters against HPV in the context of Ethiopia.

## Conclusion and recommendations

This community-based cross-sectional study conducted in Jimma Town found that the majority of caregivers expressed a willingness to have their daughters vaccinated against HPV. Caregivers with higher educational levels, good knowledge of the HPV vaccine, cervical cancer, and STIs, and positive attitudes toward the HPV vaccine were more likely to be willing to vaccinate their daughters. Provide comprehensive information to caregivers through communication channels, including mass media and parent-teacher meetings. Collaboration with NGOs that can support awareness campaigns and develop educational campaigns targeting caregivers with lower education status. Implement public health campaigns that promote positive attitudes towards the HPV vaccine by involving influential community members, respected leaders, and celebrities.

## Data Availability

The raw data supporting the conclusions of this article will be made available by the authors, without undue reservation.

## References

[1] Hall M. Mathematical modelling of human immunodeficiency virus and human papillomavirus disease transmission dynamics, natural history, and control interventions (Thesis). Sydney: UNSW Sydney library. (2021). p. 5–6. 10.26190/unsworks/22832

[2] De Martel C Plummer M Vignat J Franceschi S. Worldwide burden of cancer attributable to HPV by site, country and HPV type. Int J Cancer. (2017) 141(4):664–70. 10.1002/ijc.30716 28369882 PMC5520228

[3] Seyoum A Assefa N Gure T Seyoum B Mulu A Mihret A. Prevalence and genotype distribution of high-risk human papillomavirus infection among Sub-Saharan African women: a systematic review and meta-analysis. Front Public Health. (2022) 10:1. 10.3389/fpubh.2022.890880 PMC9304908 35875040

[4] Thomas M Narayan N Pim D Tomaić V Massimi P Nagasaka K Human papillomaviruses, cervical cancer and cell polarity. Oncogene. (2008) 27(55):7018–30. 10.1038/onc.2008.351 19029942

[5] Sung H Ferlay J Siegel RL Laversanne M Soerjomataram I Jemal A Global cancer statistics 2020: GLOBOCAN estimates of incidence and mortality worldwide for 36 cancers in 185 countries. CA Cancer J Clin. (2021) 71(3):209–49. 10.3322/caac.21660 33538338

[6] Ferlay J Ervik M Lam F Colombet M Mery L Piñeros M Global Cancer Observatory: Cancer Today. Lyon: International Agency for Research on Cancer (2020). p. 20182020.

[7] Bruni L Albero G Serrano B Mena M Collado JJ Gómez D ICO/IARC Information Centre on HPV and Cancer (HPV Information Centre). Human Papillomavirus and Related Diseases in Ethiopia. Summary Report. (2023). Available online at: https://hpvcentre.net/statistics/reports/ETH.pdf (accessed November 30, 2024).

[8] Derbie A Mekonnen D Nibret E Misgan E Maier M Woldeamanuel Y Cervical cancer in Ethiopia: a review of the literature. Cancer Causes Control. (2023) 34(1):1–11. 10.1007/s10552-022-01638-y 36242682

[9] Patanwala IY Bauer HM Miyamoto J Park IU Huchko MJ Smith-McCune KK. A systematic review of randomized trials assessing human papillomavirus testing in cervical cancer screening. Am J Obstet Gynecol. (2013) 208(5):343–53. 10.1016/j.ajog.2012.11.013 23159693 PMC3686555

[10] Erickson BK Alvarez RD Huh WK. Human papillomavirus: what every provider should know. Am J Obstet Gynecol. (2013) 208(3):169–75. 10.1016/j.ajog.2012.09.007 23021131 PMC3549042

[11] Singh D Vignat J Lorenzoni V Eslahi M Ginsburg O Lauby-Secretan B Global estimates of incidence and mortality of cervical cancer in 2020: a baseline analysis of the WHO global cervical cancer elimination initiative. Lancet Glob Health. (2023) 11(2):e197–206. 10.1016/S2214-109X(22)00501-0 36528031 PMC9848409

[12] Cheng L Wang Y Du J. Human papillomavirus vaccines: an updated review. Vaccines (Basel). (2020) 8(3):391. 10.3390/vaccines8030391 32708759 PMC7565290

[13] Gable J Eder J Noonan K Feemster K. Increasing HPV Vaccination Rates among Adolescents: Challenges and Opportunities. Philadelphia, PA: The Children’s Hospital of Philadelphia (2015).

[14] Organization WH. WHO updates Recommendations on HPV Vaccination Schedule. World Health Organization. (2023). p. 2. Available online at: https://www.who.int/news/item/20-12-2022-WHO-updates-recommendations-on-HPV-vaccination-schedule (accessed November 29, 2024).

[15] Bruni L Saura-Lazaro A Montoliu A Brotons M Alemany L Diallo MS HPV vaccination introduction worldwide and WHO and UNICEF estimates of national HPV immunization coverage 2010–2019. Prev Med. (2021) 144:106399. 10.1016/j.ypmed.2020.106399 33388322

[16] WHO-Ethiopia. Human Papillomavirus Vaccine for 14 Year Old Girls: WHO-Ethiopia. (2018). Available online at: https://www.afro.who.int/news/ethiopia-launches-human-papillomavirus-vaccine-14-year-old-girls (accessed November 28, 2024).

[17] FMOH. Health Sector Transformation Plan (HSTP 2016-2020). Addis Ababa, Ethiopia: The Federal Democratic Republic of Ethiopia Ministry of Health. (2015). p. 20–30. Available online at: https://extranet.who.int/countryplanningcycles/planning-cycle-files/ethiopia-health-sector-transformation-plan-2015-20 (accessed November 29, 2024).

[18] FMOH. Guideline for Cervical Cancer Prevention and Control in Ethiopia. Federal Democratic Republic of Ethiopia Ministry of Health. (2015). p. 9–14. Available online at: https://extranet.who.int/ncdccs/Data/ETH_D1_Cervical%20cancer%guideline-Print%version.pdf (accessed November 8, 2024).

[19] Addisu D Gebeyehu NA Belachew YY. Knowledge, attitude, and uptake of human papillomavirus vaccine among adolescent schoolgirls in Ethiopia: a systematic review and meta-analysis. BMC Women’s Health. (2023) 23(1):279. 10.1186/s12905-023-02412-1 37210492 PMC10199506

[20] WHO. Global Strategy to Accelerate the Elimination of Cervical Cancer as a Public Health Problem. Geneva, Switzerland: World Health Organization. (2020). p. 17–20. Available online at: https://www.who.int/publications-detail-redirect/9789240014107 (accessed November 28, 2024).

[21] Addisu A Getahun T Deti M Negesse Y Mekonnen B. Association of acute respiratory infections with indoor air pollution from biomass fuel exposure among under-five children in Jimma town, southwestern Ethiopia. J Environ Public Health. (2021) 2021(1):7112548. doi: 10.1155/2021/7112548 34976075 10.1155/2021/7112548 PMC8718271

[22] Ragea G Alemseged F Nigatu M Dereje D. Determinants of six-month appointment spacing model utilization among ART clients in the public health facilities of Jimma town, southwest Ethiopia: case–control study. HIV AIDS (Auckl). (2021) 13:145–56. 10.2147/HIV.S282928 33584101 PMC7874956

[23] Mihretie GN Liyeh TM Ayele AD Belay HG Yimer TS Miskr AD. Knowledge and willingness of parents towards child girl HPV vaccination in Debre Tabor town, Ethiopia: a community-based cross-sectional study. Reprod Health. (2022) 19(1):136. 10.1186/s12978-022-01444-4 35689288 PMC9188100

[24] Alene T Atnafu A Mekonnen ZA Minyihun A. Acceptance of human papillomavirus vaccination and associated factors among parents of daughters in Gondar town, northwest Ethiopia. Cancer Manag Res. (2020) 12:8519–26. 10.2147/CMAR.S275038 32982444 PMC7502398

[25] Mabeya H Odunga J Broeck DV. Mothers of adolescent girls and human papilloma virus (HPV) vaccination in western Kenya. Pan Afr Med J. (2021) 38:126. 10.11604/pamj.2021.38.126.21359 33912296 PMC8051220

[26] Adesina KT Saka A Isiaka-Lawal SA Adesiyun OO Gobir A Olarinoye AO Knowledge, practice and acceptability of HPV vaccine by mothers of adolescent girls in Ilorin, Nigeria. Sudan J Med Sci. (2018) 13(1):33–49. 10.18502/sjms.v13i1.1687

[27] Larebo YM Elilo LT Abame DE Akiso DE Bawore SG Anshebo AA Awareness, acceptance, and associated factors of human papillomavirus vaccine among parents of daughters in Hadiya Zone, Southern Ethiopia: a cross-sectional study. Vaccines (Basel). (2022) 10(12):1988. 10.3390/vaccines10121988 36560398 PMC9785952

[28] Sinshaw MT Berhe S Ayele SG. Knowledge and attitude towards human papillomavirus vaccine and associated factors among mothers who have eligible daughters in Debre Markos town, northwest Ethiopia. Infect Drug Resist. (2022) 15:781–93. 10.2147/IDR.S352440 35264861 PMC8901188

[29] Frianto D Setiawan D Diantini A Suwantika AA. Parental acceptance of human papillomavirus (HPV) vaccination in districts with high prevalence of cervical cancer in west Java, Indonesia. Patient Prefer Adherence. (2022) 16:2709–20. 10.2147/PPA.S365901 36199435 PMC9529008

[30] Mekonnen AG Bayleyegn AD Aynalem YA Adane TD Muluneh MA Zeru AB. Determinants of knowledge, attitudes, and practices in relation to HIV/AIDS and other STIs among people with disabilities in North-Shewa Zone, Ethiopia. PLoS One. (2020) 15(10):e0241312. 10.1371/journal.pone.0241312 33108410 PMC7591023

[31] Adesina KT Saka A Isiaka-Lawal SA Adesiyun OO Gobir A Olarinoye AO Parental perception of human papillomavirus vaccination of prepubertal girls in Ilorin, Nigeria. Saudi J Health Sci. (2018) 7(1):65–70. 10.4103/sjhs.sjhs_83_17

[32] Kassa HN Bilchut AH Mekuria AD Lewetie EM. Practice and associated factors of human papillomavirus vaccination among primary school students in Minjar-Shenkora district, North Shoa Zone, Amhara Regional State, Ethiopia, 2020. Cancer Manag Res. (2021) 13:6999–7008. 10.2147/CMAR.S324078 34522142 PMC8434827

[33] Onowhakpor A Omuemu V Osagie O Odili C. Human papilloma virus vaccination: knowledge, attitude and uptake among female medical and dental students in a tertiary institution in Benin-city, Nigeria. J Commun Med Primary Health Care. (2016) 28(2):101–8.

[34] Destaw A Yosef T Bogale B. Parents willingness to vaccinate their daughter against human papilloma virus and its associated factors in Bench-Sheko Zone, Southwest Ethiopia. Heliyon. (2021) 7(5):e07051. 10.1016/j.heliyon.2021.e07051 34041397 PMC8141465

[35] Dereje N Ashenafi A Abera A Melaku E Yirgashewa K Yitna M Knowledge and acceptance of HPV vaccination and its associated factors among parents of daughters in Addis Ababa, Ethiopia: a community-based cross-sectional study. Infect Agents Cancer. (2021) 16:1–7. 10.1186/s13027-021-00399-8 PMC8418033 34479576

[36] Lee K-N Chang KH-J Cho S-S Park S-H Park ST. Attitudes regarding HPV vaccinations of children among mothers with adolescent daughters in Korea. J Korean Med Sci. (2017) 32(1):130–4. 10.3346/jkms.2017.32.1.130 27914142 PMC5143285

[37] Xie Y Su L-Y Wang F Tang H-Y Yang Q-G Liu YJ. Awareness regarding and vaccines acceptability of human papillomavirus among parents of middle school students in Zunyi, Southwest China. Hum Vaccin Immunother. (2021) 17(11):4406–11. 10.1080/21645515.2021.1951931 34324411 PMC8828083

[38] Huon J-F Gregoire A Meireles A Lefebvre M Péré M Coutherut J Evaluation of the acceptability in France of the vaccine against papillomavirus (HPV) among middle and high school students and their parents. PLoS One. (2020) 15(10):e0234693 doi: 10.1371/journal.pone.0234693 33091021 10.1371/journal.pone.0234693 PMC7580947

[39] Mouallif M Bowyer H Festali S Albert A Filali Y Guenin S Primary cervical cancer prevention in Morocco: HPV vaccine awareness and acceptability among parents. Procedia Vaccinol. (2014) 8:68–76. 10.1016/j.provac.2014.07.012 24188754

[40] Azuogu B Umeokonkwo C Azuogu V Onwe O Okedo-Alex I Egbuji C. Appraisal of willingness to vaccinate daughters with human papilloma virus vaccine and cervical cancer screening uptake among mothers of adolescent students in Abakaliki, Nigeria. Niger J Clin Pract. (2019) 22(9):1286. 10.4103/njcp.njcp_452_18 31489868

[41] Cunningham MS Skrastins E Fitzpatrick R Jindal P Oneko O Yeates K Cervical cancer screening and HPV vaccine acceptability among rural and urban women in Kilimanjaro region, Tanzania. BMJ Open. (2015) 5(3):e005828. 10.1136/bmjopen-2014-005828 25757944 PMC4360576

[42] Mendes Lobão W Duarte FG Burns JD de Souza Teles Santos CA Chagas de Almeida MC Reingold A Low coverage of HPV vaccination in the national immunization programme in Brazil: parental vaccine refusal or barriers in health-service based vaccine delivery? PLoS One. (2018) 13(11):e0206726. 10.1371/journal.pone.0206726 30418980 PMC6231618

[43] Baker ML Figueroa-Downing D Chiang EDDO Villa L Baggio ML Eluf-Neto J Paving pathways: Brazil's implementation of a national human papillomavirus immunization campaign. Rev. Panam. Salud Públ. (2015) 38:163–6. 26581058

[44] Ganczak M Owsianka B Korzeń M. Factors that predict parental willingness to have their children vaccinated against HPV in a country with low HPV vaccination coverage. Int J Environ Res Public Health. (2018) 15(4):645. 10.3390/ijerph15040645 29614733 PMC5923687

[45] Chan DNS Lee PPK So WKW. Exploring the barriers and facilitators influencing human papillomavirus vaccination decisions among south Asian and Chinese mothers: a qualitative study. J Racial Ethn Health Disparities. (2024) 11(3):1465–77. 10.007/s40615-023-01623-4 37195592 PMC10191075

[46] Sitaresmi MN Rozanti NM Simangunsong LB Wahab A. Improvement of parent’s awareness, knowledge, perception, and acceptability of human papillomavirus vaccination after a structured-educational intervention. BMC public Health. (2020) 20:1–9. 10.1186/s12889-020-09962-1 33256697 PMC7708115

[47] Garcia S Shin M Sloan K Dang E Garcia CO Baezconde-Garbanati L Disruptions to and innovations in HPV vaccination strategies within safety-net healthcare settings resulting from the COVID-19 pandemic. Healthcare (Basel). (2023) 11(17):2380. doi: 10.3390/healthcare11172380 37685414 10.3390/healthcare11172380 PMC10486876

[48] Rabiu KA Alausa TG Akinlusi FM Davies NO Shittu KA Akinola OI. Parental acceptance of human papillomavirus vaccination for adolescent girls in Lagos, Nigeria. J Family Med Prim Care. (2020) 9(6):2950–7. 10.4103/jfmpc.jfmpc_102_20 32984154 PMC7491808

[49] Akinleye HW Kanma-Okafor OJ Okafor IP Odeyemi KA. Parental willingness to vaccinate adolescent daughters against human papilloma virus for cervical cancer prevention in western Nigeria. Pan Afr Med J. (2020) 36(1):4–5. doi: 10.11604/pamj.2020.36.112.9007 32821323 10.11604/pamj.2020.36.112.19007 PMC7406451

[50] Kindig DA Panzer AM Nielsen-Bohlman L. Health Literacy: A Prescription to end Confusion. Washington (DC): National Academies Press (US) (2004). p. 2–5. https://www.ncbi.nlm.nih.gov/books/NBK216035/25009856

[51] Grandahl M Chun Paek S Grisurapong S Sherer P Tyden T Lundberg P. Parents’ knowledge, beliefs, and acceptance of the HPV vaccination in relation to their socio-demographics and religious beliefs: a cross-sectional study in Thailand. PLoS One. (2018) 13(2):e0193054. 10.1371/journal.pone.0193054 29447271 PMC5814087

[52] Mansfield LN Onsomu EO Merwin E Hall NM Harper-Harrison A. Association between parental HPV knowledge and intentions to have their daughters vaccinated. West J Nurs Res. (2018) 40(4):481–501. 10.1177/0193945916682953 28322641 PMC5555819

[53] Kolek CO Opanga SA Okalebo F Birichi A Kurdi A Godman B Impact of parental knowledge and beliefs on HPV vaccine hesitancy in Kenya—findings and implications. Vaccines (Basel). (2022) 10(8):1185. 10.3390/vaccines10081185 35893833 PMC9332201

[54] Bisi-Onyemaechi AI Chikani UN Nduagubam O. Reducing incidence of cervical cancer: knowledge and attitudes of caregivers in Nigerian city to human papilloma virus vaccination. Infect Agents Cancer. (2018) 13:1–6. 10.1186/s13027-018-0202-9 PMC6098589 30140306

[55] Chaparro RM Rodríguez B Maza Y Moyano D Hernández-Vásquez A. Factors associated with hindering the acceptance of HPV vaccination among caregivers-a cross-sectional study in Argentina. PLoS One. (2020) 15(3):e0229793. 10.1371/journal.pone.0229793 32155183 PMC7064251

[56] Waser M Heiss R Borena W. Factors affecting children’s HPV vaccination in Austria: evidence from a parent survey. Hum Vaccin Immunother. (2022) 18(6):2126251. 10.1080/21645515.2022.2126251 36251011 PMC9746446

[57] Ezeanochie MC Olagbuji BN. Human papilloma virus vaccine: determinants of acceptability by mothers for adolescents in Nigeria. Afr J Reprod Health. (2014) 18(3):154–8. 25438520

