# Supplementary material for: Willingness of caregivers to have their daughters vaccinated against human papilloma virus and associated factors in Jimma Town, Southwest Ethiopia
Source: Front Glob Womens Health. 2024 Dec 12;5:1400324. doi: 10.3389/fgwh.2024.1400324 (PMC11669653; doi:10.3389/fgwh.2024.1400324)
Supplement: Supplementary file 1 [file Table1.docx]

**PART I: General background information (socio-demographic and socioeconomic characteristics)**

| S No | Variables | Category |  |  |
| --- | --- | --- | --- | --- |
|  | Age | --------- |  |  |
|  | Sex | 1. Male 2. Female |  |  |
|  | Current marital status | 1. Single 2. Married 3. Divorced 4. Widowed |  |  |
|  | Educational status | 1. No formal education 2. Primary education 3. Secondary education 4. College and above |  |  |
|  | Average monthly income | ----------------------- |  |  |
|  | The school type that the daughter enrolled | 1. Government School 2. Private school |  |  |
|  | Number of daughters aged 9-14 | 1. One 2. More than one |  |  |

**PART II Knowledge about HPV infection and HPV Vaccine**

|  | **Knowledge about HPV infection** | | |
| --- | --- | --- | --- |
| S.no | Questions | Response | Skip |
|  | Have you heard of the HPV infection? | 1. Yes 2. No | If no, go to **PART III** |
|  | Is HPV transmitted sexually? | 1. Yes 2. No 3. I don’t know |  |
|  | Is having multiple sexual partners a risk factor for HPV infection? | 1. Yes 2. No 3. I don’t know |  |
|  | Is HPV infection asymptomatic? | 1. Yes 2. No 3. I don’t know |  |
|  | Can HPV cause cervical cancer? | 1. Yes 2. No 3. I don’t know |  |
|  | Is HPV infection preventable? | 1. Yes 2. No 3. I don’t know |  |
|  | Is HPV can be prevented by abstinence from sex? | 1. Yes 2. No 3. I don’t know |  |
|  | Is HPV can be prevented by having only one sexual partner? | 1. Yes 2. No 3. I don’t know |  |
|  | **Knowledge about HPV Vaccine** | | |
| S.no | Questions | Response | Skip |
|  | Have heard of the HPV vaccine? | 1. Yes 2. No |  |
|  | Should HPV vaccination be received before sexual debut? | 1. Yes 2. No 3. I don’t know |  |
|  | A female child aged 9-14 years old offered the HPV vaccine? | 1. Yes 2. No 3. I don’t know |  |
|  | Does the HPV vaccine prevent cervical cancer? | 1. Yes 2. No 3. I don’t know |  |
|  | Does the HPV vaccine require two rounds of vaccination? | 1. Yes 2. No 3. I don’t know |  |

**PART III Knowledge about cervical cancer**

| S.no | Questions | Response | Skip |
| --- | --- | --- | --- |
|  | Have you ever heard about cervical cancer? | 1. Yes 2. No | If no, Jump to part IV |
|  | Can cervical cancer produce no signs or symptoms at an early stage? | 1. Yes 2. No 3. I don’t know |  |
|  | Is cervical cancer a preventable disease if detected early? | 1. Yes 2. No 3. I don’t know |  |
|  | Does cervical cancer risk increase in sexually active women? | 1. Yes 2. No 3. I don’t know |  |
|  | Have you ever heard about cervical cancer screening? | 1. Yes 2. No |  |
|  | Getting cervical cancer screening help for the detection of cervical cancer at an early stage? | 1. Yes 2. No 3. I don’t know |  |
|  | Cervical cancer risk can be reduced by HPV vaccination? | 1. Yes 2. No 3. I don’t know |  |

**PART IV Attitude towards HPV Vaccine**

|  | Items | Strongly  Disagree(2) | Disagree (2) | Neutral  (3) | Agree  (4) | Strongly  Agree(5) |
| --- | --- | --- | --- | --- | --- | --- |
|  | I think the HPV vaccine is safe | 1 | 2 | 3 | 4 | 5 |
|  | I think being vaccinated for HPV reduces the risk of having an HPV infection | 1 | 2 | 3 | 4 | 5 |
|  | It is best to administer the HPV vaccine before sexual initiation | 1 | 2 | 3 | 4 | 5 |
|  | My child will be fully protected after HPV vaccination | 1 | 2 | 3 | 4 | 5 |
|  | I think the HPV vaccine has Short-term side effects | 1 | 2 | 3 | 4 | 5 |
|  | I think the HPV vaccine has Unknown future side effects | 1 | 2 | 3 | 4 | 5 |

**PART V Reproductive Health-related factor**

| S.no | Questions | Response | Skip |
| --- | --- | --- | --- |
|  | Is there a history of cervical cancer either from the maternal or paternal family side? | 1. Yes 2. No |  |
|  | Do you fear that HPV could infect your daughter? | 1. Yes 2. No |  |

**Part VI Knowledge Assessment regarding STIs**

| S.no | Questions | Response | Skip |
| --- | --- | --- | --- |
|  | Have you ever heard about STIs? | 1. Yes 2. No | If no, jump to Part V |
|  | what is/are STIs | 1. Illnesses transmitted by sexual intercourse 2. Don’t know |  |
|  | Which type of STIs do you know? | 1. Gonorrhea 2. Syphilis 3. Chancroid 4. HIV/AIDS 5. Others---------- |  |
|  | Do you know the main signs/symptoms of STIs? | 1. Genital ulcer 2. Genital discharge 3. Pain during urination 4. Genital swelling 5. Others--------- |  |
|  | What are the modes of STI transmission? | 1. Sexual intercourse 2. Blood transfusion 3. Breastfeeding 4. Sharing contaminated materials 5. Other---- |  |
|  | How can one prevent STIs? | 1. Abstinence 2. Treatment of patients & their sexual partners 3. Use of condoms 4. Being faithful 5. Others ------- |  |

**PART VII Health Beliefs Regarding Cervical Cancer**

| S.no | Questions | Response | Skip |
| --- | --- | --- | --- |
| 1 | Do you think that cervical cancer is a dangerous disease? | - 1. Strongly agree(4)   2. Agree(3)   3. Disagree(2)   4. Strongly disagree(0)   5. No answer(2) |  |
| 2 | Can cervical cancer cause physical problems? | 1. Yes 2. No |  |
| 3 | Can cervical cancer cause stress? | 1. Yes 2. No |  |
| 4 | Do you worry about the possibility of cervical cancer in your family, especially children? | 1. Very worried(3) 2. Worried(2) 3. Unworried(0) 4. No answer(2) |  |

**PART VIII Husband consent**

| S.no | Questions | Response | Skip |
| --- | --- | --- | --- |
|  | Father’s approval is  mandatory for vaccination | 1. Yes 2. No |  |

**PART VX Parental willingness**

| S.no | Questions | Response | Skip |
| --- | --- | --- | --- |
| 1 | Do you know that the HPV vaccine is for daughters in our country? | 1. Yes 2. No |  |
| 2 | Are you willing to vaccinate your daughter for HPV vaccination? | 1. Yes 2. No |  |

**Thank you for your time!**
